# 1′-Allyl-1-(3,4-di­methyl­benzo­yl)-2-(4-methyl-1,3-thia­zol-5-yl)-1,2,5,6,7,7a-hexa­hydro­spiro­[pyrrolizine-3,3′-indolin]-2′-one

**DOI:** 10.1107/S1600536814006990

**Published:** 2014-04-12

**Authors:** V. Karthikeyan, V. Ramkumar, R. Joel Karunakaran

**Affiliations:** a Department of Chemistry, Madras Christian College, Tambaram, Chennai 600 059, Tamil Nadu, India; b Department of Chemistry, IIT Madras, Chennai 600 036, TamilNadu, India

## Abstract

In the title compound, C_30_H_31_N_3_O_2_S, the fused pyrrolidine ring bearing three substituents adopts an envelope conformation with the C atom bearing the benzoyl group as the flap. The other fused pyrrolidine ring adopts a twisted conformation about one of its C—C bonds. The dihedral angle between the isatin ring system and the methyl­thia­zole ring is 25.95 (8)°. An intra­molecular C—H⋯O inter­action closes an *S*(8) ring. In the crystal, mol­ecules are linked by C—H⋯O inter­actions, generating *C*(11) chains propagating in [001].

## Related literature   

For general background to spiro compounds and their biological activity, see: Pradhan *et al.* (2006 ▶); Saeedi *et al.* (2010 ▶); Dandia *et al.* (2011 ▶). For uses of oxindole derivatives, see: Rajeswaran *et al.* (1999 ▶) and of pyrrolidine derivatives, see: Suzuki *et al.* (1994 ▶). For the biological activity of pyrrolidine derivatives, see: Cuzzocrea *et al.* (2002 ▶); Obniska *et al.* (2002 ▶); Amal Raj *et al.* (2003 ▶).
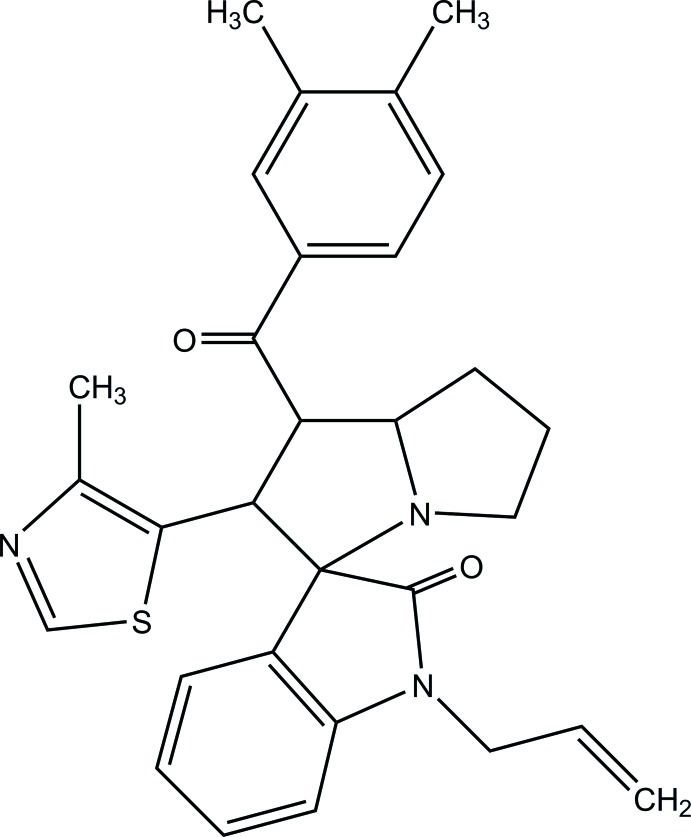


## Experimental   

### 

#### Crystal data  

C_30_H_31_N_3_O_2_S
*M* *_r_* = 497.64
Monoclinic, 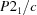

*a* = 14.5718 (4) Å
*b* = 9.7218 (2) Å
*c* = 18.2609 (5) Å
β = 94.604 (1)°
*V* = 2578.57 (11) Å^3^
*Z* = 4
Mo *K*α radiation
μ = 0.16 mm^−1^
*T* = 298 K
0.35 × 0.20 × 0.10 mm

#### Data collection  

Bruker APEXII CCD diffractometer
Absorption correction: multi-scan (*SADABS*; Bruker, 2004 ▶) *T*_min_ = 0.947, *T*_max_ = 0.984
14973 measured reflections
4494 independent reflections
3647 reflections with *I* > 2σ(*I*)
*R*_int_ = 0.017

#### Refinement  

*R*[*F* ^2^ > 2σ(*F* ^2^)] = 0.039
*wR*(*F* ^2^) = 0.112
*S* = 1.06
4494 reflections
328 parameters
H-atom parameters constrained
Δρ_max_ = 0.48 e Å^−3^
Δρ_min_ = −0.27 e Å^−3^

### 

Data collection: *APEX2* (Bruker, 2004 ▶); cell refinement: *SAINT-Plus* (Bruker, 2004 ▶); data reduction: *SAINT-Plus*; program(s) used to solve structure: *SHELXS2013* (Sheldrick, 2008 ▶); program(s) used to refine structure: *SHELXL2013* (Sheldrick, 2008 ▶); molecular graphics: *ORTEP-3 for Windows* (Farrugia, 2012 ▶); software used to prepare material for publication: *SHELXL2013*.

## Supplementary Material

Crystal structure: contains datablock(s) global, I. DOI: 10.1107/S1600536814006990/hb7204sup1.cif

Structure factors: contains datablock(s) I. DOI: 10.1107/S1600536814006990/hb7204Isup2.hkl

CCDC reference: 994375

Additional supporting information:  crystallographic information; 3D view; checkCIF report

## Figures and Tables

**Table 1:** Hydrogen-bond geometry (Å, °)

*D*—H⋯*A*	*D*—H	H⋯*A*	*D*⋯*A*	*D*—H⋯*A*
C21—H21⋯O2	0.93	2.43	3.191 (2)	138
C30—H30*A*⋯O2^i^	0.96	2.57	3.533 (3)	178

Symmetry code: (i) 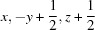.

**Fig. 1.**
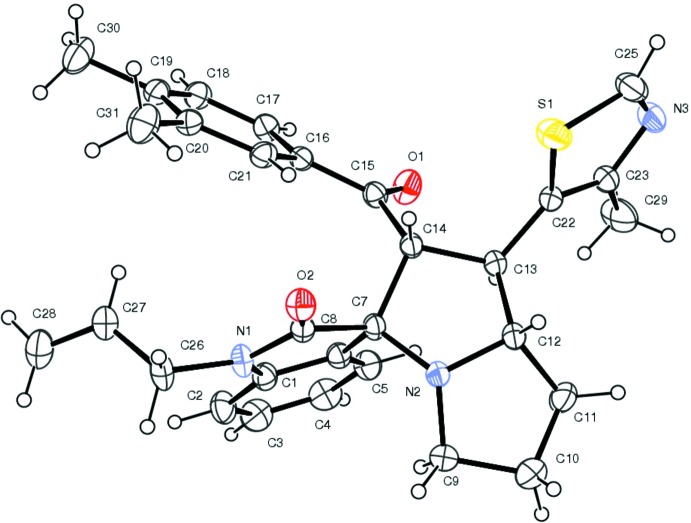
ORTEP of the molecule with atoms represented as 30% probability ellipsoids.

**Fig. 2.**
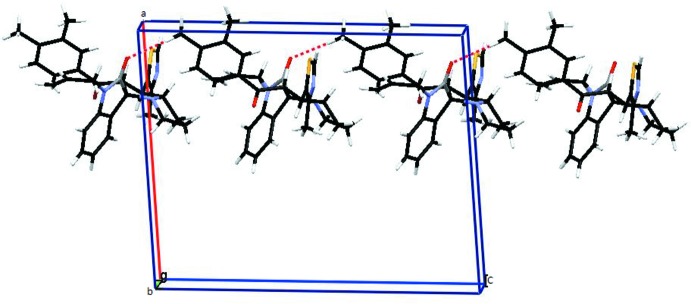
Packing diagram showing the C—H···O interaction along the C-axis

## supplementary crystallographic information

### 1. Comment

Spirooxindoles are a class of oxindoles with a 3,3-spirocyclic junction and these compounds are extremely common in nature as part of natural products as well as many synthetic drugs (Pradhan *et al.*, 2006); Oxindole derivatives help to treat and prevent diabetic complications arising from elevated levels of sorbitol, and act as aldose reductase inhibitors (Rajeswaran *et al.*,1999). Thus more and more novel spiroheterocycle compounds have been prepared and characterized (Saeedi *et al.*, 2010); Dandia *et al.*, 2011). In addition, the pyrrolidine group occurs in many families of biologically important compounds. Derivatives of pyrrolidine have anticonvulsant (Obniska *et al.*, 2002), antimicrobial and antifungal activity against various pathogens, except Bacillus subtilis (Amal Raj *et al.*, 2003). Pyrrolidine dithiocarbamate attenuates the development of acute and chronic inflammation (Cuzzocrea *et al.*, 2002). Optically active pyrrolidine derivatives have been used as intermediates in controlled asymmetric synthesis (Suzuki *et al.*, 1994). As spiro pyrrolidine compounds are of great medicinal properties, we have undertaken the three dimensional structure of the title compound. In view of these importance and continuation of our work on the crystal structure analysis of spiropyrrolidine derivatives, the crystal structure of the title compound has been carried out and the results are presented here.

The title compound, C_30_H_31_N_3_O_2_S, the pyrrolidine ring (N2/C9—C12)is twisted and the other pyrrolidine ring (N1/C1/C6—C8) is almost planar. In one of the pyrrolidine rings (N2/C9—C12) carbon atom C11 deviates by 0.537 Å from the plane. The dihedryl angle between the isatin ring and methylthiazol ring is 25.95 (8)°. The crystal structure features a C—H···O interaction which is connected along the c-axis forming a chain.

### 2. Experimental

Equimolar quantities of dimethyl acetophenone (0.02 mol) and methyl thiazole aldehyde (0.02 mol) were dissolved in 15 mL of ethanol, and aqueous NaOH (50% 12 mL) was added in dropwise. The reaction mixture was stirred at room temperature the pure compound precipitated from the solution filtered and dried. The dried thiazole aldehyde chalcone product (0.02 mol) reacted with of *L*-Proline (0.02 mol) and substituted isatins (0.02 mol) in ethanol solvent and the reaction mixture refluxed for two hours to form novel spiro compounds. The completion of the reaction monitored by TLC, the reaction mixture was cooled to room temperature and the solvent evaporated by vacuo the resulting reaction mass purified by column chromatography to isolate the pure compound. Colourless blocks of the title compound were obtained from ethanol solution by slow evaporation at room temperature.

### 3. Refinement

All hydrogen atoms were fixed geometrically and allowed to ride on the parent carbon atoms with aromatic C—H = 0.93 Å, methine C—H = 0.98 Å me thylene C—H = 0.97 Å and methyl C—H = 0.96 Å. The displacement parameters were set for phenyl H atoms at *U*_iso_(H) = 1.2*U*_eq_(C) and for methine,methylene and methyl H atoms at *U*_iso_(H) =1.5*U*_eq_(C).

### Crystal data

**Table:**

C_30_H_31_N_3_O_2_S	*F*(000) = 1056
*M**_r_* = 497.64	*D*_x_ = 1.282 Mg m^−^^3^
Monoclinic, *P*2_1_/*c*	Mo *K*α radiation, λ = 0.71073 Å
*a* = 14.5718 (4) Å	Cell parameters from 7315 reflections
*b* = 9.7218 (2) Å	θ = 2.5–28.3°
*c* = 18.2609 (5) Å	µ = 0.16 mm^−^^1^
β = 94.604 (1)°	*T* = 298 K
*V* = 2578.57 (11) Å^3^	Block, colourless
*Z* = 4	0.35 × 0.20 × 0.10 mm

### Data collection

**Table:**

Bruker APEXII CCD diffractometer	3647 reflections with *I* > 2σ(*I*)
phi and ω scans	*R*_int_ = 0.017
Absorption correction: multi-scan (*SADABS*; Bruker, 2004)	θ_max_ = 25.0°, θ_min_ = 1.4°
*T*_min_ = 0.947, *T*_max_ = 0.984	*h* = −17→17
14973 measured reflections	*k* = −11→10
4494 independent reflections	*l* = −17→21

### Refinement

**Table:**

Refinement on *F*^2^	0 restraints
Least-squares matrix: full	Hydrogen site location: inferred from neighbouring sites
*R*[*F*^2^ > 2σ(*F*^2^)] = 0.039	H-atom parameters constrained
*wR*(*F*^2^) = 0.112	*w* = 1/[σ^2^(*F*_o_^2^) + (0.0482*P*)^2^ + 1.2211*P*], where *P* = (*F*_o_^2^ + 2*F*_c_^2^)/3
*S* = 1.06	(Δ/σ)_max_ = 0.001
4494 reflections	Δρ_max_ = 0.48 e Å^−^^3^
328 parameters	Δρ_min_ = −0.27 e Å^−^^3^

### Special details

**Table:**

Geometry. All e.s.d.'s (except the e.s.d. in the dihedral angle between two l.s. planes) are estimated using the full covariance matrix. The cell e.s.d.'s are taken into account individually in the estimation of e.s.d.'s in distances, angles and torsion angles; correlations between e.s.d.'s in cell parameters are only used when they are defined by crystal symmetry. An approximate (isotropic) treatment of cell e.s.d.'s is used for estimating e.s.d.'s involving l.s. planes.

### Fractional atomic coordinates and isotropic or equivalent isotropic displacement parameters (Å^2^)

**Table:**

	*x*	*y*	*z*	*U*_iso_*/*U*_eq_	
C1	0.65413 (13)	0.39883 (19)	0.85262 (10)	0.0383 (4)	
C2	0.58768 (14)	0.4613 (2)	0.80571 (11)	0.0518 (5)	
H2	0.5986	0.5458	0.7841	0.062*	
C3	0.50416 (14)	0.3940 (3)	0.79186 (12)	0.0581 (6)	
H3	0.4582	0.4339	0.7605	0.070*	
C4	0.48832 (14)	0.2689 (2)	0.82378 (12)	0.0552 (6)	
H4	0.4323	0.2246	0.8132	0.066*	
C5	0.55540 (13)	0.2081 (2)	0.87182 (11)	0.0461 (5)	
H5	0.5441	0.1241	0.8938	0.055*	
C6	0.63861 (12)	0.27365 (18)	0.88649 (10)	0.0350 (4)	
C7	0.72224 (11)	0.23936 (17)	0.93863 (9)	0.0318 (4)	
C8	0.78884 (12)	0.35889 (17)	0.92293 (9)	0.0325 (4)	
C9	0.63441 (14)	0.3191 (2)	1.04689 (11)	0.0466 (5)	
H9A	0.5829	0.3301	1.0103	0.056*	
H9B	0.6563	0.4093	1.0629	0.056*	
C10	0.60697 (16)	0.2348 (2)	1.11132 (13)	0.0564 (6)	
H10A	0.6448	0.2578	1.1558	0.068*	
H10B	0.5428	0.2497	1.1196	0.068*	
C11	0.62314 (16)	0.0887 (2)	1.08836 (12)	0.0547 (6)	
H11A	0.5721	0.0547	1.0560	0.066*	
H11B	0.6320	0.0285	1.1307	0.066*	
C12	0.71102 (13)	0.09952 (18)	1.04812 (10)	0.0376 (4)	
H12	0.7650	0.0896	1.0833	0.045*	
C13	0.72035 (12)	0.00419 (18)	0.98251 (10)	0.0345 (4)	
H13	0.6581	−0.0167	0.9612	0.041*	
C14	0.76642 (12)	0.09562 (17)	0.92753 (9)	0.0315 (4)	
H14	0.8324	0.1013	0.9427	0.038*	
C15	0.75471 (12)	0.03912 (18)	0.84903 (10)	0.0351 (4)	
C16	0.81039 (12)	0.09678 (18)	0.79174 (9)	0.0330 (4)	
C17	0.78684 (13)	0.06349 (19)	0.71827 (10)	0.0393 (4)	
H17	0.7378	0.0045	0.7060	0.047*	
C18	0.83632 (14)	0.1180 (2)	0.66390 (10)	0.0435 (5)	
H18	0.8206	0.0938	0.6152	0.052*	
C19	0.90914 (13)	0.2083 (2)	0.68006 (10)	0.0407 (4)	
C20	0.93371 (12)	0.24228 (19)	0.75315 (10)	0.0379 (4)	
C21	0.88487 (12)	0.18377 (19)	0.80783 (10)	0.0362 (4)	
H21	0.9027	0.2036	0.8567	0.043*	
C22	0.76718 (13)	−0.12999 (18)	1.00085 (10)	0.0374 (4)	
C23	0.72805 (15)	−0.25530 (19)	1.00826 (11)	0.0456 (5)	
N3	0.78804 (15)	−0.36140 (18)	1.02743 (10)	0.0574 (5)	
C25	0.87117 (18)	−0.3172 (2)	1.03383 (12)	0.0587 (6)	
H25	0.9209	−0.3750	1.0460	0.070*	
C26	0.78500 (16)	0.5701 (2)	0.84570 (11)	0.0510 (5)	
H26A	0.8461	0.5823	0.8701	0.061*	
H26B	0.7483	0.6495	0.8569	0.061*	
C27	0.79208 (15)	0.5635 (2)	0.76434 (12)	0.0545 (6)	
H27	0.8124	0.4820	0.7445	0.065*	
C28	0.77137 (17)	0.6652 (3)	0.72003 (13)	0.0625 (6)	
H28A	0.7508	0.7480	0.7384	0.075*	
H28B	0.7771	0.6554	0.6699	0.075*	
C29	0.62774 (17)	−0.2887 (3)	0.99541 (17)	0.0746 (8)	
H29A	0.6055	−0.3229	1.0400	0.112*	
H29B	0.6191	−0.3575	0.9578	0.112*	
H29C	0.5943	−0.2072	0.9802	0.112*	
C30	0.96004 (16)	0.2666 (3)	0.61853 (12)	0.0646 (7)	
H30A	0.9340	0.2307	0.5725	0.097*	
H30B	1.0238	0.2413	0.6255	0.097*	
H30C	0.9547	0.3650	0.6183	0.097*	
C31	1.00940 (15)	0.3431 (3)	0.77401 (13)	0.0595 (6)	
H31A	1.0671	0.3062	0.7612	0.089*	
H31B	1.0123	0.3596	0.8260	0.089*	
H31C	0.9972	0.4281	0.7482	0.089*	
N1	0.74354 (10)	0.44675 (15)	0.87448 (8)	0.0393 (4)	
N2	0.70847 (10)	0.23905 (14)	1.01728 (8)	0.0353 (3)	
O1	0.69856 (10)	−0.05143 (15)	0.83445 (8)	0.0535 (4)	
O2	0.86754 (9)	0.37134 (13)	0.94943 (7)	0.0409 (3)	
S1	0.88481 (4)	−0.14445 (6)	1.01858 (3)	0.05538 (18)	

### Atomic displacement parameters (Å^2^)

**Table:**

	*U*^11^	*U*^22^	*U*^33^	*U*^12^	*U*^13^	*U*^23^
C1	0.0442 (10)	0.0379 (10)	0.0324 (10)	−0.0008 (8)	0.0013 (8)	0.0026 (8)
C2	0.0567 (12)	0.0521 (13)	0.0457 (12)	0.0046 (10)	−0.0018 (10)	0.0158 (10)
C3	0.0466 (12)	0.0744 (16)	0.0514 (13)	0.0104 (11)	−0.0084 (10)	0.0065 (12)
C4	0.0405 (11)	0.0676 (15)	0.0562 (14)	−0.0054 (10)	−0.0048 (10)	−0.0061 (11)
C5	0.0438 (10)	0.0441 (11)	0.0501 (12)	−0.0068 (9)	0.0017 (9)	−0.0002 (9)
C6	0.0379 (9)	0.0337 (10)	0.0331 (10)	−0.0010 (8)	0.0019 (7)	0.0000 (7)
C7	0.0374 (9)	0.0277 (9)	0.0303 (9)	−0.0041 (7)	0.0032 (7)	0.0019 (7)
C8	0.0420 (10)	0.0284 (9)	0.0274 (9)	−0.0042 (7)	0.0036 (7)	−0.0019 (7)
C9	0.0562 (12)	0.0392 (11)	0.0459 (12)	0.0077 (9)	0.0131 (9)	0.0006 (9)
C10	0.0591 (13)	0.0564 (14)	0.0562 (14)	0.0026 (11)	0.0204 (11)	0.0020 (11)
C11	0.0724 (14)	0.0442 (12)	0.0514 (13)	−0.0075 (10)	0.0296 (11)	0.0017 (10)
C12	0.0495 (10)	0.0308 (9)	0.0331 (10)	0.0022 (8)	0.0075 (8)	0.0047 (8)
C13	0.0407 (9)	0.0283 (9)	0.0351 (10)	−0.0003 (7)	0.0069 (8)	0.0041 (7)
C14	0.0358 (9)	0.0282 (9)	0.0308 (9)	−0.0012 (7)	0.0038 (7)	0.0027 (7)
C15	0.0398 (9)	0.0298 (9)	0.0353 (10)	−0.0014 (8)	0.0009 (8)	−0.0002 (7)
C16	0.0386 (9)	0.0292 (9)	0.0312 (9)	0.0035 (7)	0.0032 (7)	0.0004 (7)
C17	0.0459 (10)	0.0357 (10)	0.0354 (10)	−0.0010 (8)	−0.0018 (8)	−0.0015 (8)
C18	0.0546 (11)	0.0484 (12)	0.0272 (9)	0.0077 (9)	0.0013 (8)	−0.0001 (8)
C19	0.0411 (10)	0.0446 (11)	0.0372 (10)	0.0101 (9)	0.0077 (8)	0.0063 (8)
C20	0.0346 (9)	0.0397 (10)	0.0400 (11)	0.0038 (8)	0.0060 (8)	0.0023 (8)
C21	0.0393 (9)	0.0390 (10)	0.0301 (9)	0.0023 (8)	0.0023 (7)	−0.0031 (8)
C22	0.0483 (10)	0.0331 (10)	0.0321 (10)	0.0043 (8)	0.0103 (8)	0.0035 (7)
C23	0.0635 (13)	0.0310 (10)	0.0439 (11)	0.0046 (9)	0.0147 (10)	0.0032 (8)
N3	0.0843 (14)	0.0359 (10)	0.0530 (11)	0.0144 (9)	0.0116 (10)	0.0062 (8)
C25	0.0804 (17)	0.0487 (13)	0.0464 (13)	0.0279 (12)	0.0009 (12)	0.0014 (10)
C26	0.0656 (13)	0.0370 (11)	0.0497 (12)	−0.0152 (10)	0.0008 (10)	0.0130 (9)
C27	0.0633 (13)	0.0458 (13)	0.0562 (14)	−0.0030 (10)	0.0160 (11)	0.0126 (10)
C28	0.0760 (16)	0.0611 (15)	0.0510 (14)	−0.0116 (12)	0.0082 (12)	0.0139 (11)
C29	0.0684 (16)	0.0407 (13)	0.117 (2)	−0.0092 (11)	0.0203 (15)	0.0028 (14)
C30	0.0615 (14)	0.0898 (19)	0.0442 (13)	−0.0014 (13)	0.0154 (11)	0.0134 (12)
C31	0.0477 (12)	0.0712 (16)	0.0601 (14)	−0.0150 (11)	0.0069 (11)	0.0050 (12)
N1	0.0468 (9)	0.0334 (8)	0.0368 (9)	−0.0089 (7)	−0.0016 (7)	0.0093 (7)
N2	0.0459 (8)	0.0285 (8)	0.0323 (8)	0.0006 (6)	0.0078 (7)	0.0018 (6)
O1	0.0681 (9)	0.0484 (9)	0.0446 (8)	−0.0245 (7)	0.0081 (7)	−0.0083 (6)
O2	0.0408 (7)	0.0399 (7)	0.0413 (7)	−0.0082 (6)	−0.0012 (6)	−0.0010 (6)
S1	0.0504 (3)	0.0509 (3)	0.0646 (4)	0.0101 (2)	0.0029 (3)	0.0022 (3)

### Geometric parameters (Å, º)

**Table:**

C1—C2	1.381 (3)	C15—C16	1.484 (2)
C1—C6	1.392 (3)	C16—C21	1.389 (2)
C1—N1	1.411 (2)	C16—C17	1.396 (2)
C2—C3	1.387 (3)	C17—C18	1.379 (3)
C2—H2	0.9300	C17—H17	0.9300
C3—C4	1.376 (3)	C18—C19	1.390 (3)
C3—H3	0.9300	C18—H18	0.9300
C4—C5	1.391 (3)	C19—C20	1.394 (3)
C4—H4	0.9300	C19—C30	1.506 (3)
C5—C6	1.377 (3)	C20—C21	1.393 (3)
C5—H5	0.9300	C20—C31	1.502 (3)
C6—C7	1.522 (2)	C21—H21	0.9300
C7—N2	1.466 (2)	C22—C23	1.357 (3)
C7—C8	1.555 (2)	C22—S1	1.7244 (19)
C7—C14	1.559 (2)	C23—N3	1.379 (3)
C8—O2	1.214 (2)	C23—C29	1.497 (3)
C8—N1	1.362 (2)	N3—C25	1.282 (3)
C9—N2	1.468 (2)	C25—S1	1.716 (2)
C9—C10	1.514 (3)	C25—H25	0.9300
C9—H9A	0.9700	C26—N1	1.459 (2)
C9—H9B	0.9700	C26—C27	1.499 (3)
C10—C11	1.506 (3)	C26—H26A	0.9700
C10—H10A	0.9700	C26—H26B	0.9700
C10—H10B	0.9700	C27—C28	1.298 (3)
C11—C12	1.530 (3)	C27—H27	0.9300
C11—H11A	0.9700	C28—H28A	0.9300
C11—H11B	0.9700	C28—H28B	0.9300
C12—N2	1.468 (2)	C29—H29A	0.9600
C12—C13	1.529 (3)	C29—H29B	0.9600
C12—H12	0.9800	C29—H29C	0.9600
C13—C22	1.498 (2)	C30—H30A	0.9600
C13—C14	1.535 (2)	C30—H30B	0.9600
C13—H13	0.9800	C30—H30C	0.9600
C14—C15	1.532 (2)	C31—H31A	0.9600
C14—H14	0.9800	C31—H31B	0.9600
C15—O1	1.217 (2)	C31—H31C	0.9600
			
C2—C1—C6	121.97 (18)	C21—C16—C17	118.18 (16)
C2—C1—N1	128.02 (18)	C21—C16—C15	122.89 (16)
C6—C1—N1	110.01 (15)	C17—C16—C15	118.93 (16)
C1—C2—C3	117.8 (2)	C18—C17—C16	120.03 (18)
C1—C2—H2	121.1	C18—C17—H17	120.0
C3—C2—H2	121.1	C16—C17—H17	120.0
C4—C3—C2	120.95 (19)	C17—C18—C19	121.61 (18)
C4—C3—H3	119.5	C17—C18—H18	119.2
C2—C3—H3	119.5	C19—C18—H18	119.2
C3—C4—C5	120.6 (2)	C18—C19—C20	119.08 (17)
C3—C4—H4	119.7	C18—C19—C30	119.51 (18)
C5—C4—H4	119.7	C20—C19—C30	121.41 (19)
C6—C5—C4	119.3 (2)	C21—C20—C19	118.87 (17)
C6—C5—H5	120.4	C21—C20—C31	119.46 (17)
C4—C5—H5	120.4	C19—C20—C31	121.65 (17)
C5—C6—C1	119.34 (17)	C16—C21—C20	122.17 (17)
C5—C6—C7	131.98 (17)	C16—C21—H21	118.9
C1—C6—C7	108.61 (15)	C20—C21—H21	118.9
N2—C7—C6	116.59 (14)	C23—C22—C13	128.09 (18)
N2—C7—C8	108.57 (13)	C23—C22—S1	109.12 (14)
C6—C7—C8	101.61 (13)	C13—C22—S1	122.77 (14)
N2—C7—C14	102.47 (13)	C22—C23—N3	115.7 (2)
C6—C7—C14	115.69 (14)	C22—C23—C29	126.31 (19)
C8—C7—C14	112.06 (14)	N3—C23—C29	117.93 (19)
O2—C8—N1	126.11 (16)	C25—N3—C23	110.32 (19)
O2—C8—C7	125.75 (15)	N3—C25—S1	115.74 (17)
N1—C8—C7	108.14 (14)	N3—C25—H25	122.1
N2—C9—C10	104.31 (16)	S1—C25—H25	122.1
N2—C9—H9A	110.9	N1—C26—C27	112.65 (17)
C10—C9—H9A	110.9	N1—C26—H26A	109.1
N2—C9—H9B	110.9	C27—C26—H26A	109.1
C10—C9—H9B	110.9	N1—C26—H26B	109.1
H9A—C9—H9B	108.9	C27—C26—H26B	109.1
C11—C10—C9	103.73 (17)	H26A—C26—H26B	107.8
C11—C10—H10A	111.0	C28—C27—C26	123.7 (2)
C9—C10—H10A	111.0	C28—C27—H27	118.2
C11—C10—H10B	111.0	C26—C27—H27	118.2
C9—C10—H10B	111.0	C27—C28—H28A	120.0
H10A—C10—H10B	109.0	C27—C28—H28B	120.0
C10—C11—C12	102.96 (16)	H28A—C28—H28B	120.0
C10—C11—H11A	111.2	C23—C29—H29A	109.5
C12—C11—H11A	111.2	C23—C29—H29B	109.5
C10—C11—H11B	111.2	H29A—C29—H29B	109.5
C12—C11—H11B	111.2	C23—C29—H29C	109.5
H11A—C11—H11B	109.1	H29A—C29—H29C	109.5
N2—C12—C13	105.10 (14)	H29B—C29—H29C	109.5
N2—C12—C11	104.56 (15)	C19—C30—H30A	109.5
C13—C12—C11	117.70 (16)	C19—C30—H30B	109.5
N2—C12—H12	109.7	H30A—C30—H30B	109.5
C13—C12—H12	109.7	C19—C30—H30C	109.5
C11—C12—H12	109.7	H30A—C30—H30C	109.5
C22—C13—C12	114.85 (15)	H30B—C30—H30C	109.5
C22—C13—C14	115.80 (14)	C20—C31—H31A	109.5
C12—C13—C14	103.44 (14)	C20—C31—H31B	109.5
C22—C13—H13	107.4	H31A—C31—H31B	109.5
C12—C13—H13	107.4	C20—C31—H31C	109.5
C14—C13—H13	107.4	H31A—C31—H31C	109.5
C15—C14—C13	112.50 (14)	H31B—C31—H31C	109.5
C15—C14—C7	115.27 (14)	C8—N1—C1	111.42 (14)
C13—C14—C7	103.13 (13)	C8—N1—C26	123.80 (16)
C15—C14—H14	108.6	C1—N1—C26	124.68 (15)
C13—C14—H14	108.6	C7—N2—C12	112.11 (13)
C7—C14—H14	108.6	C7—N2—C9	121.47 (15)
O1—C15—C16	120.99 (16)	C12—N2—C9	110.19 (14)
O1—C15—C14	119.13 (16)	C25—S1—C22	89.08 (11)
C16—C15—C14	119.87 (15)		
			
C6—C1—C2—C3	−1.1 (3)	C15—C16—C17—C18	178.67 (16)
N1—C1—C2—C3	179.66 (19)	C16—C17—C18—C19	−1.1 (3)
C1—C2—C3—C4	−0.1 (3)	C17—C18—C19—C20	1.3 (3)
C2—C3—C4—C5	1.1 (4)	C17—C18—C19—C30	−179.07 (19)
C3—C4—C5—C6	−0.8 (3)	C18—C19—C20—C21	0.5 (3)
C4—C5—C6—C1	−0.4 (3)	C30—C19—C20—C21	−179.18 (18)
C4—C5—C6—C7	175.96 (19)	C18—C19—C20—C31	−177.71 (19)
C2—C1—C6—C5	1.4 (3)	C30—C19—C20—C31	2.6 (3)
N1—C1—C6—C5	−179.25 (17)	C17—C16—C21—C20	2.6 (3)
C2—C1—C6—C7	−175.73 (18)	C15—C16—C21—C20	−176.85 (16)
N1—C1—C6—C7	3.6 (2)	C19—C20—C21—C16	−2.5 (3)
C5—C6—C7—N2	−63.4 (3)	C31—C20—C21—C16	175.78 (18)
C1—C6—C7—N2	113.23 (17)	C12—C13—C22—C23	101.5 (2)
C5—C6—C7—C8	178.8 (2)	C14—C13—C22—C23	−137.95 (19)
C1—C6—C7—C8	−4.58 (18)	C12—C13—C22—S1	−76.69 (19)
C5—C6—C7—C14	57.1 (3)	C14—C13—C22—S1	43.9 (2)
C1—C6—C7—C14	−126.20 (16)	C13—C22—C23—N3	−178.37 (17)
N2—C7—C8—O2	60.6 (2)	S1—C22—C23—N3	0.0 (2)
C6—C7—C8—O2	−175.91 (17)	C13—C22—C23—C29	3.7 (3)
C14—C7—C8—O2	−51.8 (2)	S1—C22—C23—C29	−178.0 (2)
N2—C7—C8—N1	−119.33 (15)	C22—C23—N3—C25	−0.5 (3)
C6—C7—C8—N1	4.11 (18)	C29—C23—N3—C25	177.7 (2)
C14—C7—C8—N1	128.21 (15)	C23—N3—C25—S1	0.8 (2)
N2—C9—C10—C11	−32.1 (2)	N1—C26—C27—C28	136.2 (2)
C9—C10—C11—C12	37.9 (2)	O2—C8—N1—C1	177.76 (17)
C10—C11—C12—N2	−29.4 (2)	C7—C8—N1—C1	−2.3 (2)
C10—C11—C12—C13	−145.56 (18)	O2—C8—N1—C26	1.3 (3)
N2—C12—C13—C22	152.65 (15)	C7—C8—N1—C26	−178.70 (17)
C11—C12—C13—C22	−91.5 (2)	C2—C1—N1—C8	178.46 (19)
N2—C12—C13—C14	25.51 (18)	C6—C1—N1—C8	−0.8 (2)
C11—C12—C13—C14	141.35 (17)	C2—C1—N1—C26	−5.1 (3)
C22—C13—C14—C15	72.1 (2)	C6—C1—N1—C26	175.59 (18)
C12—C13—C14—C15	−161.31 (14)	C27—C26—N1—C8	115.9 (2)
C22—C13—C14—C7	−163.02 (15)	C27—C26—N1—C1	−60.1 (3)
C12—C13—C14—C7	−36.48 (17)	C6—C7—N2—C12	108.89 (17)
N2—C7—C14—C15	156.57 (14)	C8—C7—N2—C12	−137.19 (15)
C6—C7—C14—C15	28.6 (2)	C14—C7—N2—C12	−18.49 (18)
C8—C7—C14—C15	−87.22 (17)	C6—C7—N2—C9	−24.4 (2)
N2—C7—C14—C13	33.57 (16)	C8—C7—N2—C9	89.54 (19)
C6—C7—C14—C13	−94.39 (17)	C14—C7—N2—C9	−151.77 (16)
C8—C7—C14—C13	149.78 (14)	C13—C12—N2—C7	−4.19 (19)
C13—C14—C15—O1	13.8 (2)	C11—C12—N2—C7	−128.77 (16)
C7—C14—C15—O1	−104.05 (19)	C13—C12—N2—C9	134.38 (16)
C13—C14—C15—C16	−167.26 (15)	C11—C12—N2—C9	9.8 (2)
C7—C14—C15—C16	74.9 (2)	C10—C9—N2—C7	147.77 (17)
O1—C15—C16—C21	−169.49 (18)	C10—C9—N2—C12	13.7 (2)
C14—C15—C16—C21	11.6 (3)	N3—C25—S1—C22	−0.64 (18)
O1—C15—C16—C17	11.0 (3)	C23—C22—S1—C25	0.32 (15)
C14—C15—C16—C17	−167.86 (15)	C13—C22—S1—C25	178.81 (16)
C21—C16—C17—C18	−0.8 (3)		

### Hydrogen-bond geometry (Å, º)

**Table:**

*D*—H···*A*	*D*—H	H···*A*	*D*···*A*	*D*—H···*A*
C21—H21···O2	0.93	2.43	3.191 (2)	138
C30—H30*A*···O2^i^	0.96	2.57	3.533 (3)	178

Symmetry code: (i) *x*, −*y*+1/2, *z*+1/2.

## References

[bb1] Amal Raj, A., Raghunathan, R., Sridevi Kumari, M. R. & Raman, N. (2003). *Bioorg. Med. Chem.* **11**, 407–409. 10.1016/s0968-0896(02)00439-x 12517436

[bb2] Bruker (2004). *APEX2, *SAINT-Plus** and *SADABS* Bruker AXS Inc., Madison, Wisconsin, USA.

[bb3] Cuzzocrea, S., Chatterjee, P. K., Mazzon, E., Dugo, L., Serraino, I., Britti, D., Mazzullo, G., Caputi, A. P. & Thiemermann, C. (2002). *Br. J. Pharmacol.* **135**, 496–510. 10.1038/sj.bjp.0704463 PMC1573136 11815386

[bb4] Dandia, A., Singh, R., Bhaskarana, S. & Samant, S. D. (2011). *Green Chem.* **13**, 1852–1859.

[bb5] Farrugia, L. J. (2012). *J. Appl. Cryst.* **45**, 849–854.

[bb6] Obniska, J., Zeic, A. & Zagorska, A. (2002). *Acta Pol. Pharm.* **59**, 209–213. 12230248

[bb7] Pradhan, R., Patra, M., Behera, A. K. & Behera, R. K. (2006). *Tetrahedron*, **62**, 779–828.

[bb8] Rajeswaran, W. G., Labroo, R. B. & Cohen, L. A. (1999). *J. Org. Chem.* **64**, 1369–1371.

[bb9] Saeedi, M., Heravi, M. M., Beheshtiha, Y. S. & Oskooie, H. A. (2010). *Tetrahedron*, **66**, 5345–5348.

[bb10] Sheldrick, G. M. (2008). *Acta Cryst.* A**64**, 112–122. 10.1107/S0108767307043930 18156677

[bb11] Suzuki, H., Aoyagi, S. & Kibayashi, C. (1994). *Tetrahedron Lett.* **35**, 6119–6122.

